# Impact of Nematode Infections on Non-specific and Vaccine-Induced Humoral Immunity in Dual-Purpose or Layer-Type Chicken Genotypes

**DOI:** 10.3389/fvets.2021.659959

**Published:** 2021-05-11

**Authors:** Gürbüz Daş, Monika Auerbach, Manuel Stehr, Christian Sürie, Cornelia C. Metges, Matthias Gauly, Silke Rautenschlein

**Affiliations:** ^1^Institute of Nutritional Physiology, Leibniz Institute for Farm Animal Biology (FBN), Dummerstorf, Germany; ^2^Clinic for Poultry, University of Veterinary Medicine Hannover, Hanover, Germany; ^3^Farm for Education and Research, University of Veterinary Medicine Hannover, Hanover, Germany; ^4^Faculty of Science and Technology, Free University of Bozen-Bolzano, Bolzano, Italy

**Keywords:** ascarids, animal health, alternative to culling, host genetic background, immunity, vaccination

## Abstract

Nematode infections may induce immune-modulatory effects and influence host-immune responses to other pathogens. The aim of the study was to investigate whether a mixed nematode-infection influences non-specific and vaccine-induced humoral immunity against Newcastle Disease Virus (NDV), Infectious Bronchitis Virus (IBV), and Avian Metapneumovirus (AMPV) in already vaccinated hens of a dual-purpose (Lohmann Dual, LD) or a layer genotype (Lohmann Brown Plus; LB). Until 17 weeks-of-age, LD (*n* = 70) and LB (*n* = 109) hens were vaccinated against major bacterial and viral diseases and coccidiosis. At 24 weeks-of-age, the hens received either a placebo or an oral inoculation of 1,000 infectious eggs of *A. galli* and *H. gallinarum*. Plasma total immunoglobulin (Ig) isotypes (IgY, IgM, IgA) levels and vaccine-induced antibody titers against NDV, IBV, and AMPV were determined from 2 to 18 weeks post-infection (wpi). Infections had no suppressing effect on total Ig isotypes IgY, IgM, and IgA as well as on vaccine-induced antibody titers against NDV, IBV, and AMPV (*P* > 0.05). Overall, LB hens had higher levels of IgY, IgM, and IgA than those of LD hens (*P* < 0.05). There were no differences between IBV titers of the two genotypes (*P* > 0.05). Independent of infection status of the hens, NDV titers were higher in LB hens than in LD hens at wpi 2 (*P* < 0.05), but not in following weeks (*P* > 0.05). Uninfected LD hens had lower AMPV titers than their infected counterparts at 6 and 14 wpi (*P* < 0.05). Regardless of nematode infection, LD hens revealed a higher risk of responding weak to vaccination against NDV (odds ratio = 5.45; *P* = 0.021) and AMPV (odds ratio = 13.99, *P* < 0.001) than did LB hens (*P* > 0.05). We conclude that nematode infections have no adverse effects on non-specific and vaccine-induced humoral immunity in either genotype. LB hens have higher levels of total immunoglobulin isotypes than LD hens. Except for IBV, vaccine-induced humoral immune responses show a dependency on genotype. Dual-purpose hens show lower responsiveness to vaccinations against NDV and AMPV, possibly due to factors associated with increased body fat reserves in this genotype.

## Introduction

Modern chicken genotypes that are used for commercial egg or meat production have been genetically selected for one-way production mode only, i.e., egg or meat production. This is mainly because of the strong genetic antagonism between reproduction and growth traits in chickens (1). In other words, because egg production is a sex-dependent phenotype for which layer genotypes are strongly selected for, poor growth performance and efficiency of male birds of the layer lines are not competitive to that of meat-type chickens. As a consequence in many countries, male birds are killed as day-old-birds for economic reasons (2, 3). The killing of day-old birds has however lead to extensive debates in Europe (4, 5), and alternatives to culling of the male birds of layer lines are currently being extensively explored. As reviewed by Krautwald-Junghanns et al. (6), in-ovo sexing techniques have a great potential to be used as a tool. However, an automatized and commercially viable use of in-ovo sex determination techniques is not widely available yet. One of the available potential alternatives to culling of male birds is the reintegration of the so-called dual-purpose genotypes in production systems in order to use female birds for egg production, while counterpart males are used for meat production (1, 6). Recent studies have clearly demonstrated that the use of dual-purpose genotypes is associated with compromises of both egg and meat production (7–9). Despite the lower performance of dual-purpose genotypes as compared to specialized layer or meat-type genotypes, their implementation in farming systems might nevertheless contribute to the mitigation of high-performance associated health and welfare problems in both broilers and laying hens (10, 11). Furthermore, dual-purpose genotypes may need lower protein levels in their diets, implying a smaller dependency on the highly nutrient dense diets for high performance birds (12, 13). Whether dual-purpose genotypes have improved health and welfare associated traits remain largely unknown.

From the most recent studies focusing on the comparison of both male and female birds of a dual-purpose genotype with commercial meat or layer-type chickens, we draw the main conclusion that high performing chickens are less tolerant to nematode infections (8, 9). Resistance to nematode infections, however, depends on both host genotype as well as on the nematode species involved in the mixed infections (8, 9). We focused on nematode infections as they constitute a concrete problem in the field, particularly in non-cage housing systems (14–17). The most commonly encountered nematodes are *Heterakis gallinarum* followed by *Ascaridia galli* (14, 15, 18, 19). Helminth infections may modulate the immune system, which may interfere with the immune response to other pathogens in the same host (20). Thus, there have been discussions on whether helminth-infected chickens are more vulnerable to infections with intra-cellular pathogens, including viruses and bacteria. Chicken's immune system deals with intracellular (e.g., viruses) and extracellular (e.g., nematodes) pathogens mainly through the Th1- and Th2-type immune responses, respectively (21, 22). This implies that nematode-infected chickens may be more vulnerable to intracellular pathogens. A critical argument supporting this hypothesis came from a study by Degen et al. (21) who demonstrated that Th-1 and Th2-type immune responses maybe traded off in chickens infected with Newcastle Disease Virus (NDV) or *A. galli*, respectively. In line with this, naturally helminth-infected local hens that received an anthelmintic treatment showed higher antibody titers following vaccination against NDV than their non-treated helminth-infected counterparts (23). A more recent study by Pleidrup et al. (20) demonstrated that chickens infected with *A. galli* exhibited impaired humoral and cell-mediated immune responses after vaccination against NDV.

To our knowledge, it is unknown whether vaccine-induced humoral immunity to viral pathogens are hampered in nematode-infected hens that are routinely vaccinated during the growing period. Because host-animal performance level is associated with tolerance to nematode infections in chickens (8, 9), nematode-infected chickens with high or lower performance levels may mount different immune responses to vaccinations against other pathogens. Therefore, the aim of this study was to investigate the effects of nematode-infection on vaccine-induced specific humoral- immunity against viral pathogens, including NDV, Infectious Bronchitis Virus (IBV) and Avian Metapneumovirus (AMVP), as well as on total immunoglobulin isotypes in routinely vaccinated hens of high or lower performing genotypes. A dual-purpose genotype with lower laying performance was particularly included in the study in order to further investigate whether such genotypes have different health-associated properties than hens of high performing genotypes.

## Materials and Methods

### Ethics Statement

Ethical approval of the experiment was obtained from the relevant state ethics committees for animal experimentations (Lower Saxony State Office for Consumer Protection and Food Safety, Germany, Permission no.: 33.19-42502-05-15A594; Mecklenburg-Western Pomerania State Office for Agriculture, Food Safety, and Fisheries, Germany; permission no.: 7221.3-1-080/16). The experiment was conducted in accordance with animal welfare rules (animal care and handling, stunning, necropsies) and all sampling procedures were performed by trained/authorized staff. Experimental infection procedures were in line with the relevant guidelines of the World Association for the Advancement of Veterinary Parasitology for Poultry (24).

### Hens and Vaccination Program

The study included samples from a total of 179 hens of two genotypes, namely Lohmann Brown Plus (LB; *n* = 109) and Lohmann Dual (LD; *n* = 70). During the growing period (17 weeks), the hens were subjected to a conventional vaccination program that included immunization against major bacterial and viral diseases as well as coccidiosis at recommended ages (Table 1). Except for vaccinations at d0, all vaccinations were applied at the Farm for Education and Research in Ruthe, University of Veterinary Medicine Hannover. Vaccinations were performed as recommended by the manufacturers. During the growing period in which the vaccination program was also applied, the birds of the two genotypes were raised under same husbandry conditions in separate housing units of the same facility. Following the last vaccinations, the pullets were transported to the Experimental Poultry Facility of Leibniz Institute for Farm Animal Biology (FBN) at around of 17 weeks of age.

**Table 1:** Summary of the conventional vaccination program applied to Lohmann Brown Plus and Lohmann Dual hens during growing period (17 weeks).

**Age, d**	**Vaccination against**
0	MDV + IBD + IBV
1	*Salmonella enteritidis* + *S. typhimurium*
7	Coccidiosis
24	NDV
30^*^	IBV
44	NDV
50	*Salmonella enteritidis* + *S. typhimurium*
57	*Mycoplasma gallisepticum*
63	Avian pneumovirus-ART (AMPV)
72	IBV
78	NDV
85	Avian encephalomyelitis
113	*Salmonella enteritidis* + *S. typhimurium*
119	IBV + NDV + EDS + ART (AMPV)

*MDV, Marek's Disease Virus; IBD, Infectious Bursal Disease; IBV, Infectious Bronchitis Virus; NDV, Newcastle Disease Virus; ART, Avian pneumovirus—ART (i.e., AMPV: Avian Metapneumovirus); EDS, Egg Drop Syndrome*.

* *originally planned for d16, but post-poned to d30*.

### Experimental Design, Nematode Infection, and Environmental Conditions

The experimental design of the study was a 3-factorial arrangement of treatments with nematode infection (infected vs. control), host genotype (LB vs. LD), and time as weeks post-infection (wpi 2–18). Following the arrival of the pullets at the FBN, all birds of each genotype were given individual wing-tags, and were randomly allocated to one of 12 pens in two adjacent rooms, each equipped with 6 pens, in the same facility. After the entry into the laying phase (i.e., laying rate >50%), all hens in the first room (i.e., 3 pens per genotype) were experimentally infected with embryonated eggs of two nematodes (see below for infection procedures), while all the hens in 6 pens (i.e., 3 pens per genotype) of the second room were kept as uninfected controls. Number of hens per genotype in each pen varied from 6 to 23 hens with a fixed stock density of maximum 6 hens per m^2^.

An experimental (co-)infection with *A. galli* and *H. gallinarum* was induced when the hens were 24-wk old. Nematode eggs used as the infection material were collected from worms of naturally infected free-range chickens. Incubation conditions for embryonation of the nematode eggs and the preparation of the final infection inoculum have been described in detail elsewhere (25). The percentage of incubated, fully embryonated eggs considered infectious was determined (26). On the day of infection, separately incubated eggs of *A. galli* and *H. gallinarum* were merged to a final dosage of 0.4 ml/hen containing a total of 1,000 embryonated eggs of the two species in equal proportions (1:1, i.e., 500 eggs per worm species). All hens in the first room were given a single infection dose orally by using a 5-cm esophageal cannula. Starting from 2 weeks post-infection (wpi), i.e., at the age of 26 weeks, infected and uninfected hens of both genotypes were randomly collected from each pen and necropsied at timed intervals (i.e., 2, 4, 6, 10, 14, and 18 wpi) to quantify infection intensity with either nematode. The length of the infection experiment (18 weeks) was planned to assess hens' responses to both primary (experimental) and subsequently occurring natural re-infections. Total number of hens necropsied at each wpi ranged from 29 to 34. Infection intensity (i.e., worm burdens) with either nematode was quantified as described earlier (9). In brief, the small intestine was opened longitudinally and the intestinal content was washed through a sieve (36 μm) under running water to collect mature and immature stages of *A. galli* from the intestinal lumen. The tissue-associated *A. galli* larvae were recovered by using a modified EDTA (10 mM EDTA, 0.9% NaCl)-incubation method for digesting intestinal tissue overnight at 40°C, followed by sieving (25, 27, 28). *H. gallinarum* was harvested from the caecal lumen contents only as described for *A. galli* from the small intestine. The caecal tissue and lumen contents were flushed under running water through a sieve (20 μm) to isolate immature and mature *H. gallinarum*. Worms of both species collected from each chicken were then counted using a stereo microscope.

In the pre-experimental period (24 weeks), the hens were kept in helminth-free conditions. During the experimental period, the hens received no vaccinations or medical treatments, including anthelminthics. During the experimental period, the hens were fed a commercial laying-hen diet that contained 11.2 MJ metabolizable energy, 170 g crude protein and 3.6 g Calcium per kg feed (i.e., as-fed basis). Feed and water were offered for *ad libitum* intake. Lighting and temperature regimes were as suggested by the breeding company. The hens were kept under floor husbandry conditions using wood shavings as litter material. Litter was not removed until the end of the experiment.

### Measurement of Vaccine-Induced Antibodies and Immunoglobulin Isotypes

For the present study we used blood samples from 179 hens (*n* = 109 LB and *n* = 70 LD). Slaughter blood was collected from the hens in potassium-EDTA (Kabe Labortechnik GmbH, Nümbrecht-Elsenroth, Germany). Blood was centrifuged at 2,500 g for 20 min, and the supernatant was stored at −20°C for later analysis.

Plasma concentrations of vaccine-induced antibodies against Newcastle Disease Virus (NDV), Infectious Bronchitis Virus (IBV) and Avian Metapneumovirus (AMVP), were analyzed by commercially available ELISAs following the protocols recommended by the manufacturers (ND Synbiotics ProFLOK+®, IB Synbiotics ProFLOK®, Synbiotics Corporation, San Diego, CA, USA: Avian Rhinotracheitis Antibody test kit; BioChek, Reeuwijk, NL).

Commercial ELISA Kits (IgY: Kit No. E30-104; IgM: Kit No. E30-103; IgA: Kit No. E30-102; Bethyl Laboratories, Inc, Montgomery, TX, USA) were used to analyse immunoglobulin concentrations (IgY, IgM, IgA) in EDTA-plasma samples (*N* = 175). ELISAs were performed according to the manufacturer's instructions. A pooled plasma sample served as a control among all plates. The laboratory-specific intra-assay CV and inter-assay CV for the analysis ranged between 5.0 and 7.6% and 7.7 and 10.4%, respectively.

### Statistical Analyses

The experimental unit was a hen for the statistical analysis of all variables presented in this study. The vaccine-induced antibody titers and Ig data were subjected to analysis of variance by using the GLM procedure in the SAS/STAT (Version 9.4) software of the SAS System for Windows (SAS Institute Inc., Cary, NC, USA). The statistical model included fixed effects of host genotype, nematode infection, wpi and all possible interactions among these three factors, plus blocking effect of the pens.

Least-squares means (LSM) and their standard errors (SE) were computed for each fixed effect in the model, and all pairwise differences in these LSMs were tested with the Tukey-Kramer correction for multiple comparisons. In addition, the SLICE statement of the MIXED procedure was used for performing partitioned analyses of the LSMs for the two- or three-way interactions (e.g., test of infection within the levels of wpi in each genotype). Effects and differences were considered significant at *P* ≤ 0.05, or tended to be significant 0.05 < *P* ≤ 0.10. Unless a significant interaction between the effects of investigated factors was present, results were presented only for the main effects in tables or figures. In case of significant interactions, results were additionally illustrated in figures.

Pearson correlations between body weight (BW), IgY, IgA, IgM, and antibody titers against NDV, IBV, and AMPV were calculated for each genotype separately, using genotype-pooled data across different weeks post-infection.

In a further analysis, responsiveness to vaccination was evaluated statistically using the distribution of the respective antibody-titer data. For this purpose, distribution of the respective antibody-titer data were used to classify birds as responder or weak-responder to vaccination. Birds which did not show vaccine-induced antibody titers within the range of 2 SD below the overall mean of a particular antibody titer were considered as weak-responders (i.e., mean titer−2 SD > weak-responder) for that particular vaccination. Frequencies of the weak-responder and responder birds (i.e., mean titer-−2 SD ≤ responder) were then calculated. Potential effects of relevant factors on the probability of being a weak-responder to vaccination were quantified using a Generalized Estimating Equations (GEE) logistic regression model with a logit link function using GLIMMIX procedure of SAS software. The statistical model included the fixed effects of host genotype, nematode infection, wpi, and the interaction between genotype and infection, whereas interactions of genotype and infections with wpi were excluded, as the number of weak-responder vaccinees for the combination of the three factors were limited. Odds ratios (Ψ) were calculated for all main effects included in the logistic regression model.

## Results

Parasitological parameters describing fecal egg counts, worm burdens and nematode-specific antibody levels of the two genotypes are presented in details elsewhere (9). In brief, there was no significant difference between LB and LD hens in terms of number of eggs per gram feces (EPG) at any time point (*P* > 0.05). All experimentally nematode infected hens harbored worm(s), ranging from a sum of 1–573 worms of both species per hen. On average each hen harbored 14 (SD =13.5) *A. galli* and 139 (SD =103) *H. gallinarum*. The overall average *A. galli* and *H. gallinarum* counts, which were based on the sum of the all juvenile and mature stages of the worms, did not significantly differ between the two genotypes (*P* > 0.05), whereas LB hens had higher levels of reinfection with *H. gallinarum* than did LD hens (*P* < 0.05). The levels of ascarid-specific IgY levels in both plasma and egg-yolks were also higher in LB than in LD following experimental and subsequent secondary (naturally occurring) reinfections. Both fecal egg counts and parasitological examination of the intestines confirmed infection free status of the uninfected control animals in both genotypes.

**Figure 1:**
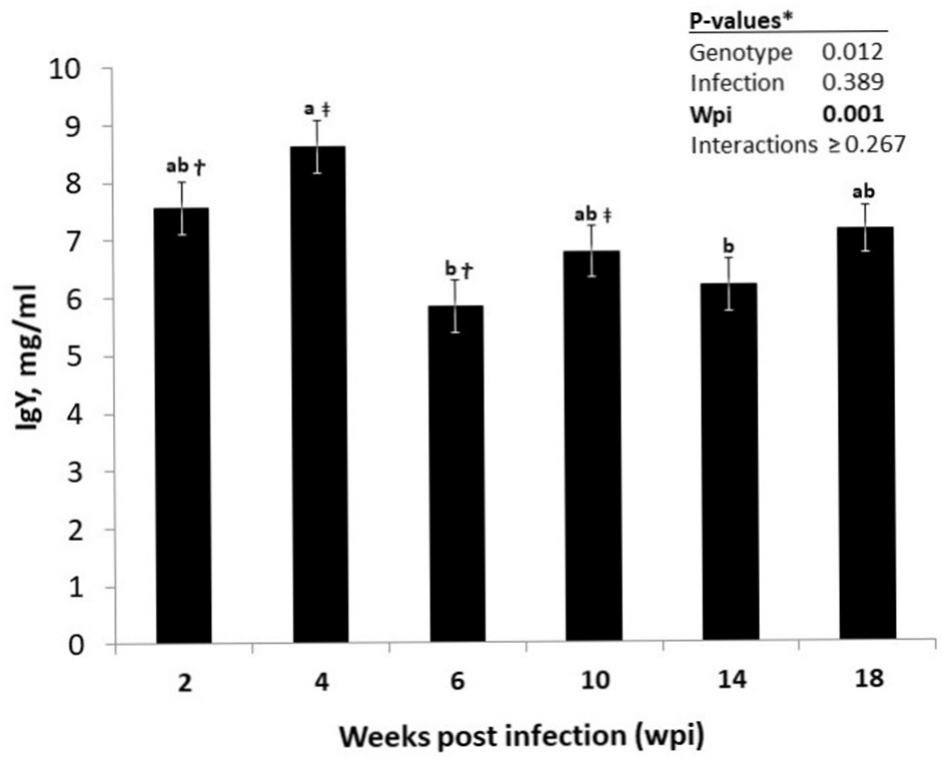
Time-dependent alterations in the course of circulating IgY levels in infected or uninfected hens of Lohmann Brown Plus and Lohmann Dual genotypes over the experimental period. *For the significant genotype differences as well for the non-significant *P-*values for all possible interaction effects see Table 2. ^ab^Different letters indicate significant differences between different time points (*p* < 0.05). ^†^Time points (wpi) sharing the sign tend to differ (*p* = 0.086). ^‡^Time points (wpi) sharing the sign tend to differ (*p* = 0.052).

Plasma concentrations of total immunoglobulin isotypes of the hens strongly depended on the host genotype (Table 2), with LB hens having higher levels of circulating IgY (*P* = 0.012), IgM (*P* < 0.001), and IgA (*P* < 0.001) than those of the LD hens. No effects of the nematode infection on the immunoglobulin concentrations were quantified, either as the main effect or as the interaction with wpi or host genotype (*P* > 0.05). Time had a significant effect on IgY levels of the hens (*P* < 0.001; Figure 1) without an interaction with host genotype or infection or both (*P* > 0.05). The IgY levels of the hens, irrespective of their genotype or infection statues, were higher at wpi 4 than at wpi 6 and 14 (*P* < 0.05). Furthermore, the IgY levels tended to be higher at wpi 4 than in wpi 10 (*p* = 0.052), as well as at wpi 2 than in wpi 6 (*p* = 0.086).

**Table 2:** Concentrations of total immunoglobulin isotypes, IgY, IgM, and IgA in plasma samples of laying hens of Lohmann Brown Plus (LB) and Lohmann Dual (LD) genotypes exposed to an experimental mixed-nematode-infection (Inf.) or kept as uninfected control (Con.).

	**Genotype**				**Nematode infection**				**Further effects (*****P-*****values**, **≤)**				
**Item**	**LD**	**LB**	**SE**	**P,≤**	**Con**.	**Inf**.	**SE**	**P,≤**	**wpi**	Inf.*Gen.	Inf.*wpi	Gen.*wpi	Inf.*Gen.*wpi
IgY, mg/ml	6.56	7.49	0.288	0.012	6.87	7.18	0.287	0.389	0.001	0.877	0.267	0.564	0.887
IgM, mg/ml	0.63	0.76	0.027	0.001	0.69	0.69	0.027	0.806	0.916	0.344	0.249	0.947	0.792
IgA, mg/ml	0.27	0.33	0.009	0.001	0.29	0.30	0.009	0.547	0.082	0.286	0.170	0.268	0.467

**Figure 2:**
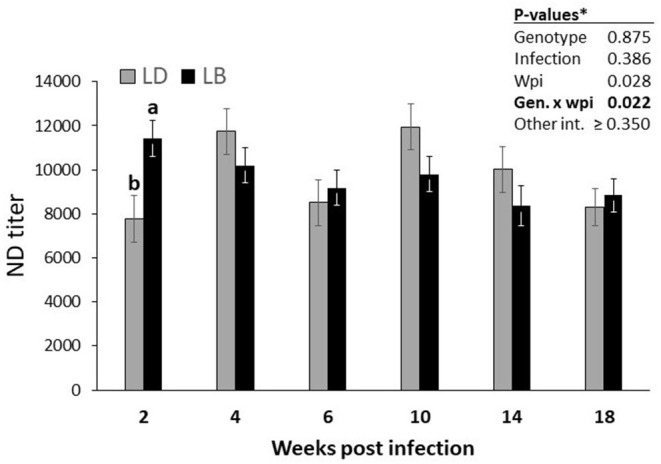
Time-dependent alterations in the course of vaccine-induced NDV titers in Lohmann Brown Plus (LB) and Lohmann Dual (LD) genotypes over the experimental period. ^*^Exact *P-*values for all possible interaction effects are presented in Table 2. ^ab^Different letters between two genotypes indicate significant differences at the same wpi (*p* < 0.05).

The two genotypes did not differ in IBV titers (Table 3; *P* = 0.112). Similarly, nematode infection induced no effect on the IBV titers (*P* = 0.810). IBV titers of the hens did not change significantly throughout the experimental weeks (*P* = 0.177). Furthermore, no significant interaction effects among the three main factors were quantified (*P* > 0.05). Nematode infection did not influence NDV titers of the hens of either genotype at any time point (*P* = 0.386; Table 3). A significant interaction of genotype by time (*P* = 0.022) indicated higher NDV titers in LB hens than in LD hens at wpi 2 (*P* < 0.05; Figure 2). In the following weeks, no other significant differences were found between two genotypes (*P* > 0.05). A triple interaction between the effects of genotype, infection and wpi was not observed on the NDV titers (*P* = 0.350).

**Table 3:** Vaccine-induced antibody levels against Infectious Bronchitis Virus (IBV), Newcastle Disease virus (NDV), and avian Metapneumovirus (AMVP) in laying hens in relation to host genotype and mixed-nematode infection.

	**Genotype**				**Nematode infection**				**Further effects (*****P-*****values**, **≤)**				
**Item**	**LD**	**LB**	**SE**	**P,≤**	**Con**.	**Inf**.	**SE**	**P,≤**	**wpi**	Inf.*Gen.	Inf.*wpi	Gen.*wpi	Inf.*Gen.*wpi
IBV titer	13,986	16,030	1,011	0.112	14,854	15,162	1,012	0.810	0.177	0.131	0.401	0.874	0.210
NDV titer	9,716	9,633	419	0.875	9,444	9,905	420	0.386	0.028	0.631	0.652	0.022	0.350
AMPV titer	20,122	25,551	921	0.001	21,616	24,057	916	0.038	0.115	0.162	0.083	0.222	0.024

**Figure 3:**
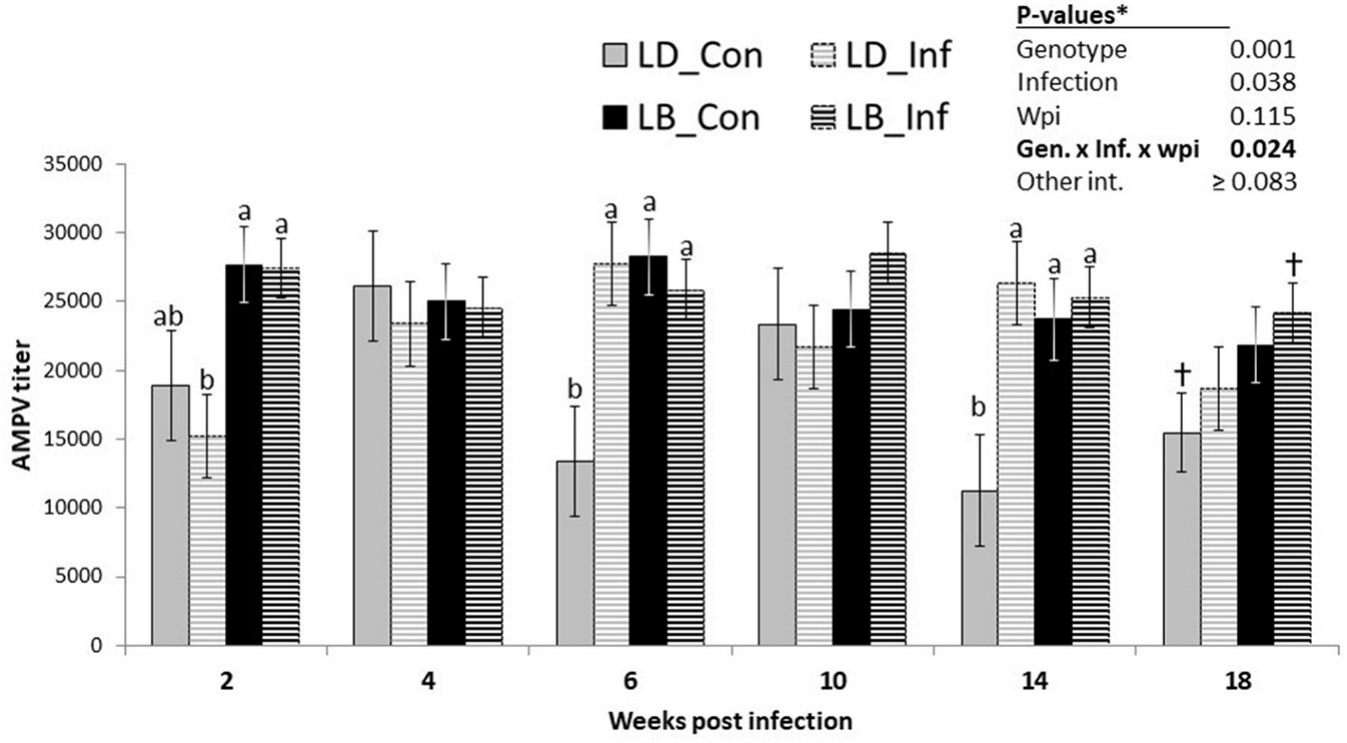
Course of vaccine-induced Avian Metapneumovirus (AMVP) titers in nematode-uninfected (_Con) or infected (_Inf) hens of Lohmann Brown Plus (LB) and Lohmann Dual (LD) genotypes over a period of 18 weeks post-infection. ^ab^Different letters indicate significant differences among 4 groups at a given wpi (*p* < 0.05). ^†^Groups sharing the sign tend to differ (*p* = 0.068).

On average, LB hens had higher AMPV titers than LD hens (Table 3; *P* < 0.001). Similarly, infected hens had higher levels of AMPV antibodies than their uninfected counterparts (*P* = 0.038). However, as revealed by the significant interaction among the effects of genotypes, infection and time, the differences between infected and uninfected hens of the two genotypes were shown to be time dependent (Figure 3; *P* = 0.024). At wpi 2, infected LD hens had lower AMPV titers than LB hens with or without infection (*P* < 0.05). At both wpi 6 and wpi 14, uninfected LD hens had lower AMPV titers than the hens of other three groups (*P* < 0.05). At wpi 18, uninfected LD hens tended to have lower AMPV titers than infected LB hens (*P* = 0.068).

**Figure 4:**
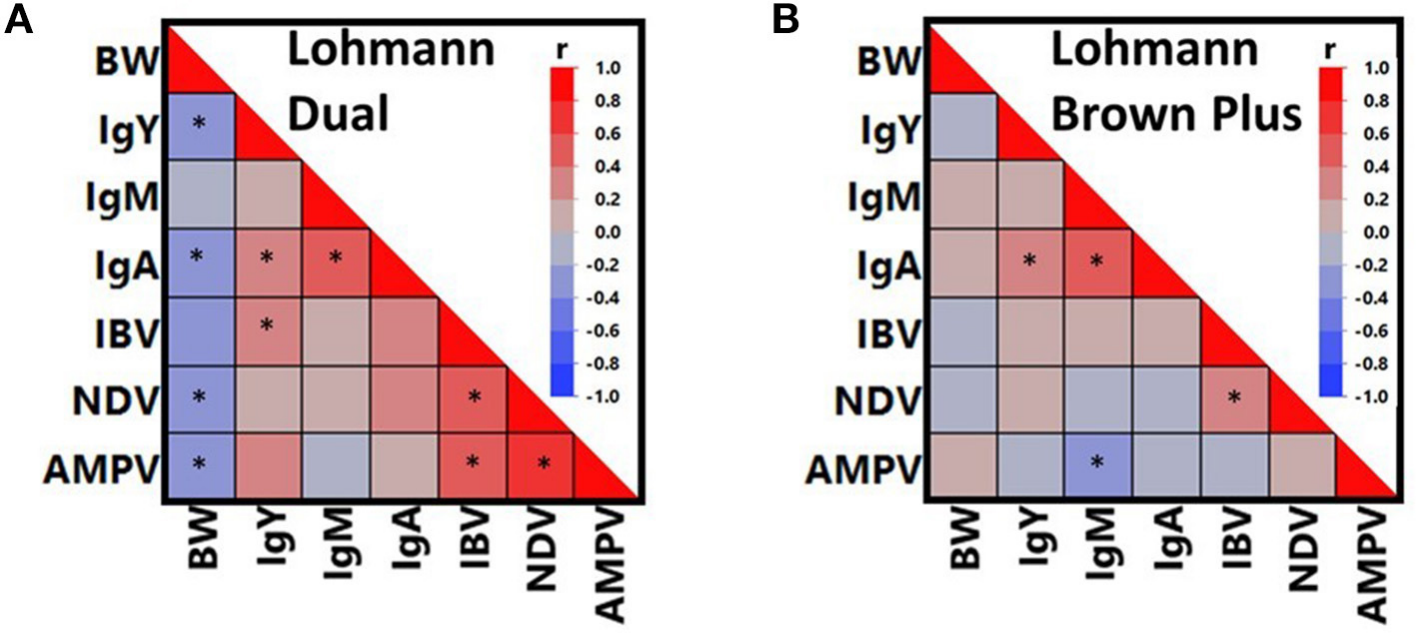
Color maps presenting correlations between body weight (BW), total immunoglobulin isotypes (IgY, IgM, IgA) and vaccine-induced antibody titers against Infectious Bronchitis Virus (IBV), Newcastle Disease Virus (NDV), and Avian Metapneumovirus (AMPV) in Lohmann Dual **(A)** and Lohmann Brown Plus **(B)** chicken genotypes. *The sign indicates a significant correlation at *p* < 0.05.

Figure 4 presents all possible correlations between total Ig isotypes, vaccine-induced humoral immune responses and BW in both genotypes, separately. As confirmed within either genotype, IgA correlated positively with IgY and IgM (*P* < 0.05), whereas no correlation was found between IgY and IgM (*P* > 0.05). Similarly, significant positive correlations were calculated between IBV and NDV titers in both genotypes (*P* < 0.05). Although the relationship between IgM and AMPV titer appeared to be inverse in both genotypes, a significant correlation was found only in LB hens (*P* < 0.05).

There were additional relationships, which were specific to only LD hens (Figure 4). For instance, BW correlated negatively with IgY, IgA, NDV and AMPV (*p* < 0.05). On the contrary, AMPV titers were positively correlated with IBV and NDV titers (*P* < 0.05). Although IgY and IBV titers correlated positively in LD hens (*P* < 0.05), this relationship was also absent in LB hens (*P* > 0.05).

Responsiveness to IBV vaccination was 100%, and antibody titers were detected in all birds irrespective of genotype or nematode infection status. In contrast to the case with IBV, a total of 6.8 and 9.1% of the hens were weak-responders to NDV and AMPV vaccinations, respectively. As presented in Table 4, a higher percentage of LD hens were weak-responders to vaccinations with NDV (12.9%) and AMPV (20.6%) as compared to LB hens. Probability of being weak-responder to NDV was 4.45 times (i.e., Ψ = 5.45) higher in LD than in LB hens (*P* = 0.021). When compared to LB hens, the LD hens were ~13 times more likely to be weak-responder to vaccination against AMPV (*P* < 0.001). Approximately half the weak-responder LD hens were simultaneously weak-responders to both NDV or AMPV (Supplementary Figure 1).

**Table 4:** Frequency (%) and odds ratios (Ψ) assessing the probability of being weak-responder to vaccination against Newcastle Disease Virus (NDV), and Avian Metapneumovirus (AMVP) in relation to host genotype, nematode infection, and weeks post-infection (wpi) in laying hens.

**Factors**		**NDV**		**AMPV**	
		**%**	**Ψ**	**%**	**Ψ**
Genotype	LD	12.9	5.45	20.6	13.99
	LB	2.8	1.00	1.9	1.00
	*P-value*	*0.021*		*0.001*	
Nematode infection	Uninfected	7.3	0.94	13.2	1.97
	Infected	6.5	1.00	6.5	1.00
	*P-value*	*0.931*		*0.387*	
Genotype x infection	*P-value*	*0.808*		*0.803*	
Weeks post-infection	wpi 2	13.8	1.37	13.4	1.09
	wpi 4	0.0	0.01	6.9	0.45
	wpi 6	6.9	0.61	6.9	0.45
	wpi 10	0.0	0.01	3.5	0.20
	wpi 14	7.4	0.62	7.4	0.48
	wpi 18	11.8	1.00	15.6	1.00
	*P-value*	*0.953*		*0.663*	

Nematode infection did not influence the probability of weak-responding to any vaccination (*P* ≥ 0.387; Table 4). There were also no interaction between the effects of host genotype and nematode infection on the probability of weak-responding to vaccination against NDV or AMPV (*P* > 0.05). Similarly, probability of being weak-responder to vaccinations did not depend on time after infection (*P* > 0.05).

## Discussion

We investigated the effects of nematode infections and host-genotype on total immunoglobulin isotypes and vaccine-induced specific antibodies of vaccinated hens for a period of 18 weeks following experimental infections. Our results collectively suggest that nematode infections have no adverse effects on non-specific or vaccine-induced humoral immune responses in already vaccinated chickens. Hens of the dual-purpose genotype had lower levels of all immunoglobulin isotypes than did the LB hens, but both genotypes developed and maintained similar levels of vaccine induced antibody titers against IBV for a relatively long period of time. Uninfected LD hens had temporary lower AMPV titers than those of LB hens, although nematode infection did not aggravate this effect. We identified a sub-cluster of LD hens, which were particularly under risk as weak-responder to vaccinations against NDV and AMPV. An increased body weight in LD hens was associated with lower antibody titers against NDV and AMPV. In the following sections, we address potential mechanisms underlying differences between nematode infected and uninfected vaccinated hens of the dual-purpose (LD) and the layer-type (LB) chicken genotypes in response to common antiviral vaccinations.

### Non-specific Immunoglobulins

According to Koenen et al. (29), meat-type chickens show a lower cytokine response but a strong short-term humoral immune response, whereas layers rely on a strong cellular response accompanied by a long-term humoral immune response. LB hens had higher concentrations of immunoglobulin isotypes (i.e., IgY, IgM, IgA) than the LD hens. These differences might reflect, at least partly, different types of immune programming in broiler and layer type chickens, which corresponds to their productive lifespans. Differences in the immunoglobulin patterns of chickens with respect to breeding objectives was investigated previously in male birds of LB, LD and Ross-308 genotypes during the first 10 weeks of life (8). When compared with broilers, LB birds had higher IgY but lower IgM levels, whereas dual-purpose birds were more closer to LB than to Ross. The overall concentrations of both IgY and IgM in young birds were however approximately the half of the mature hens in the present study, implying that immunoglobulin concentration is age-dependent (30). A sex-dependent difference between immunoglobulin levels of LB and LD genotypes can however not be ruled out. Although nematode-infected male birds of both LB and LD genotypes had higher levels of IgY and IgM than their uninfected counterparts (8), there was no significant difference in immunoglobulin levels of the hens induced by the nematode infections. These results are in agreement with data presented by Saasa et al. (31), who found no difference between antibody titers to sheep red blood cells in dewormed or un-dewormed chickens that were naturally infected with helminths.

### Vaccine-Induced Antibodies

Under commercial conditions, pullets of layer type chickens receive a series of vaccinations against viral, bacterial and protozoon pathogens during the growing period (e.g., Table 1). As shown previously, pronounced differences exist between layer and meat type chickens in innate and adaptive immune responses following vaccination (32) and infection with IBD (33), implying the necessity of genotype specific vaccination regimes. We measured humoral immune responses to vaccination against IBV, NDV and AMPV in both nematode-infected and uninfected hens of two genotypes. The results suggest that nematode infections have no adverse effects on total Ig isotypes or vaccine-induced humoral immune responses in vaccinated chickens of either genotype, though genotype effects appear to be more important.

Our results may appear to be in contradiction with previous results demonstrating that nematode-infected animals have impaired vaccine-induced humoral immune responses against NDV (20, 23). However, there are crucial differences between designs of the present and previous studies. One of the main differences between the present and previous studies, which may have caused the different outcome, is that we first vaccinated the hens and thereafter infected them with nematodes. By using the order of “vaccination > infection” we intended to mimic commercial farming conditions, where animals generally receive immunizations against several pathogens other than nematodes during the growing period, and thereafter encounter nematodes usually much later when they are in production farms. In contrast to our study, in both previous studies (20, 23) chickens were first naturally or experimentally infected and thereafter received vaccination against NDV. It is likely that the order of nematode infection and vaccination can influence the outcome of the immune responses. The study of Pleidrup et al. (20) reports an interesting result that *A. galli* infected hens had lower vaccine-induced NDV titers as compared to their *A. galli*-uninfected but ND-vaccinated counterparts. There was however no difference in NDV-titers between *A. galli* infected and uninfected animals that were not NDV-vaccinated but challenged with the NDV. This observation implies that if chickens are first exposed to NDV-vaccination and then nematode-infection, their response to vaccination may differ from the case if they would be infected with the nematode first and thereafter vaccinated against NDV. This is indeed reasonable as immunomodulatory properties of nematodes may particularly affect the effector phase of vaccine responses (20), whereas in vaccinated animals that have already established vaccine-induced immune responses impact of nematode infection appears to be insignificant. Thus, the order of vaccination and nematode-infections appears to play an important role in producing antibody response to vaccination, and it should particularly be investigated in future studies.

Irrespective of the nematode infection, LB hens had higher NDV titers than LD hens 2 weeks after infection, implying an age- but not infection-dependent difference between the two genotypes in generating and/or maintain an immune response to vaccination against NDV. In general, LB hens had higher AMPV titers than LD hens (Table 3), but this difference was not constant, as it was shown to be both time and infection dependent (Figure 3). Although there was no difference in AMPV titers of infected and uninfected LD hens at 2 wpi, infected LD hens had lower AMPV titers as compared with infected or uninfected hens of LB genotype. In contrast to this, uninfected LD hens had lower AMPV titers than infected counterpart birds of the same genotype at wpi 6 and 14. We do not have an explanation why uninfected LD hens necropsied at these two time points showed lower AMPV titers than did uninfected LD and both infected or uninfected LB hens. Nevertheless, these differences occurred at the two time points, when IgY levels of all hens were at the lowest level (Figure 1), implying a general stress likely associated with increasing laying performance.

It is crucially important to note that we used the term “weak-responders” for animals that did not show antibody titers within the range of 2 SD below the overall mean antibody titers measured 9–25 weeks after the last vaccinations. This definition excludes the possibility that animals classified as weak-responders might initially have well-responded to vaccination, but failed to maintain the level of antibody titers at the time of blood sampling, i.e., > 9 weeks post-vaccination. A possible failure could likely be due to the combination of factors related to quality of the humoral response (e.g., memory cell induction) and the short half-life of IgY antibodies, which ranges from 36 to 65 h in chickens (34). Nevertheless, both genotypes were subjected to the same classification, thus responsiveness to vaccination allows a relative comparison of the two genotypes in terms of maintaining their antibody titers long time after vaccinations. The results indicated that responsiveness to vaccination against both NDV and AMPV was genotype dependent, with a higher percentage of LD hens being classified as weak-responders than those of LB hens (Table 4). As compared with LD hens, the LB hens were ~4.5–13 times more likely to respond to vaccinations with elevated levels of NDV and AMPV antibody titers, respectively. It is interesting to note that about half of the weak-responder LD hens did not respond to both NDV and AMPV (Supplementary Figure 1). As elaborated above, layer and broiler type chickens differ in their immune programming (29). The dual-purpose genotype used in the present study is a combination of layer and broiler lines, and takes an intermediate position between layer and meat type chickens for most growth parameters (8) and immunological responses after vaccination (32). There is however a large heterogeneity in the body weight of LD birds. Urban et al. (12) identified a distinct bimodal distribution in body weight of male birds of the dual-purpose genotype, which may imply an incomplete fixation of the genome for growth related parameters. Whether such body-weight related differences have a link to immune responsiveness within the dual-purpose genotype deserves further detailed investigations.

Overall, per capita egg mass production is lower in LD hens than in LB hens. Furthermore, LD hens have lower body weights than LB hens (9), due to existence of a sex-linked dwarf gene, which has major suppressing effects on body weights only in females of LD genotype (1). Despite the smaller body size, *ad libitum* consumption of conventional layer diets increases body fat content in LD hens (35). Indeed, Röhe et al. (35) found a positive correlation between BW and body fat content, whereas egg production was negatively correlated with body fat content in LD hens. In the present study, correlation analysis revealed remarkable relationships of BW with non-specific and vaccine-induced humoral immune responses in LD hens, which were absent in LB hens. Body weight in LD hens correlated negatively with IgY, IgA and antibody titers against NDV and AMPV, implying the higher BW the lower most humoral immune responses in LD hens. This finding is in line with vaccine failures and increased susceptibility to infections in obese people, likely due to immunosuppressive effects of fat tissue that produces different cytokines and adipokines, which interact with T and B cell receptors (36). In agreement with this, different studies confirm an elevated risk of being non-responder to hepatitis B vaccination in health workers (37) and in hepatitis B virus naïve women (38), suggesting obesity-associated factors interfere with vaccine immunogenicity (38).

All vaccine-induced humoral immune responses were positively correlated in LD hens, whereas only NDV and IBV titers correlated in LB hens. These positive correlations in LD hens may indicate that the individuals that are able to successfully generate and maintain an immune response following vaccination against one pathogen, will also do so with vaccination against another pathogen. In turn, LD birds that cannot mount or maintain an immune response after vaccination against one pathogen, will not be able to do so against another pathogen, too. In line with the observations of Urban et al. (12) on the bimodal distribution of body weight in LD genotype, the differences in the patterns of relationships between humoral immune responses and body weight in LD and LB birds indicate that LD hens are not as uniform as the LB hens in terms of developing immune responses to vaccinations. Considering the presence of a larger group of weak-responders to vaccinations against NDV and AMPV in LD than in LB genotypes, and negative associations between BW and vaccine-induced immune responses in LD hens, it is reasonable to postulate that adaptive immune responses of LD hens might have unintentionally been traded-off with growth-traits through the genetic selection and crossbreeding procedures. This hypothesis is in line with the outcome of a meta-analysis by van der Most et al. (39) that confirms compromised immune functions due to selection for growth in poultry. In the particular case of the LD genotype, a more reasonable explanation might be that an increased body weight associated with higher body fat content compromises immune function (36). As shown in the present study, dual-purpose genotypes are not necessarily superior to high performing genotypes in terms of non-specific and vaccine-induced immune responses. This implies that dual-purpose genotypes that could potentially contribute to prevention of culling of male birds as well as to the mitigation of high-performance associated health and welfare problems, should also be genetically improved for immune functions.

## Conclusion

Our results collectively suggest that nematode infections induce no adverse effects on non-specific and vaccine-induced humoral immune responses in already vaccinated hens of either genotype. LB hens have higher levels of total immunoglobulin isotypes than LD hens. Except for IBV, vaccine-induced humoral immune responses show a dependency on genotype. Dual-purpose hens show lower responsiveness to vaccinations against NDV and AMPV, likely due to factors associated with increased body fat reserves in this genotype.

## Data Availability Statement

The raw data supporting the conclusions of this article will be made available by the authors, without undue reservation.

## Ethics Statement

The animal study was reviewed and approved by Lower Saxony State Office for Consumer Protection and Food Safety, Germany, Permission no.: 33.19-42502-05-15A594; Mecklenburg-Western Pomerania State Office for Agriculture, Food Safety, and Fisheries, Germany; permission no.: 7221.3-1-080/16.

## Author Contributions

GD, MG, and SR obtained funding. GD, MG, CCM, and SR conceived and designed the study. GD, MA, MS, CS, and SR contributed to acquisition, rearing, vaccination, infection, and sampling of the hens and/or performed other works related to conducting the animal experiment. GD, MA, MS, and SR contributed to the analyses of the samples and collection of data. GD performed statistical analysis of the data and drafted the manuscript. MA, MG, MS, CS, CCM, and SR reviewed the manuscript. All authors read the article and approved the submitted version.

## Conflict of Interest

The authors declare that the research was conducted in the absence of any commercial or financial relationships that could be construed as a potential conflict of interest.
