# Supplementary material for: Impact of Nematode Infections on Non-specific and Vaccine-Induced Humoral Immunity in Dual-Purpose or Layer-Type Chicken Genotypes
Source: Front Vet Sci. 2021 May 11;8:659959. doi: 10.3389/fvets.2021.659959 (PMC8144313; doi:10.3389/fvets.2021.659959)
Supplement: Supplementary file 1 [file Data_Sheet_1.PDF]

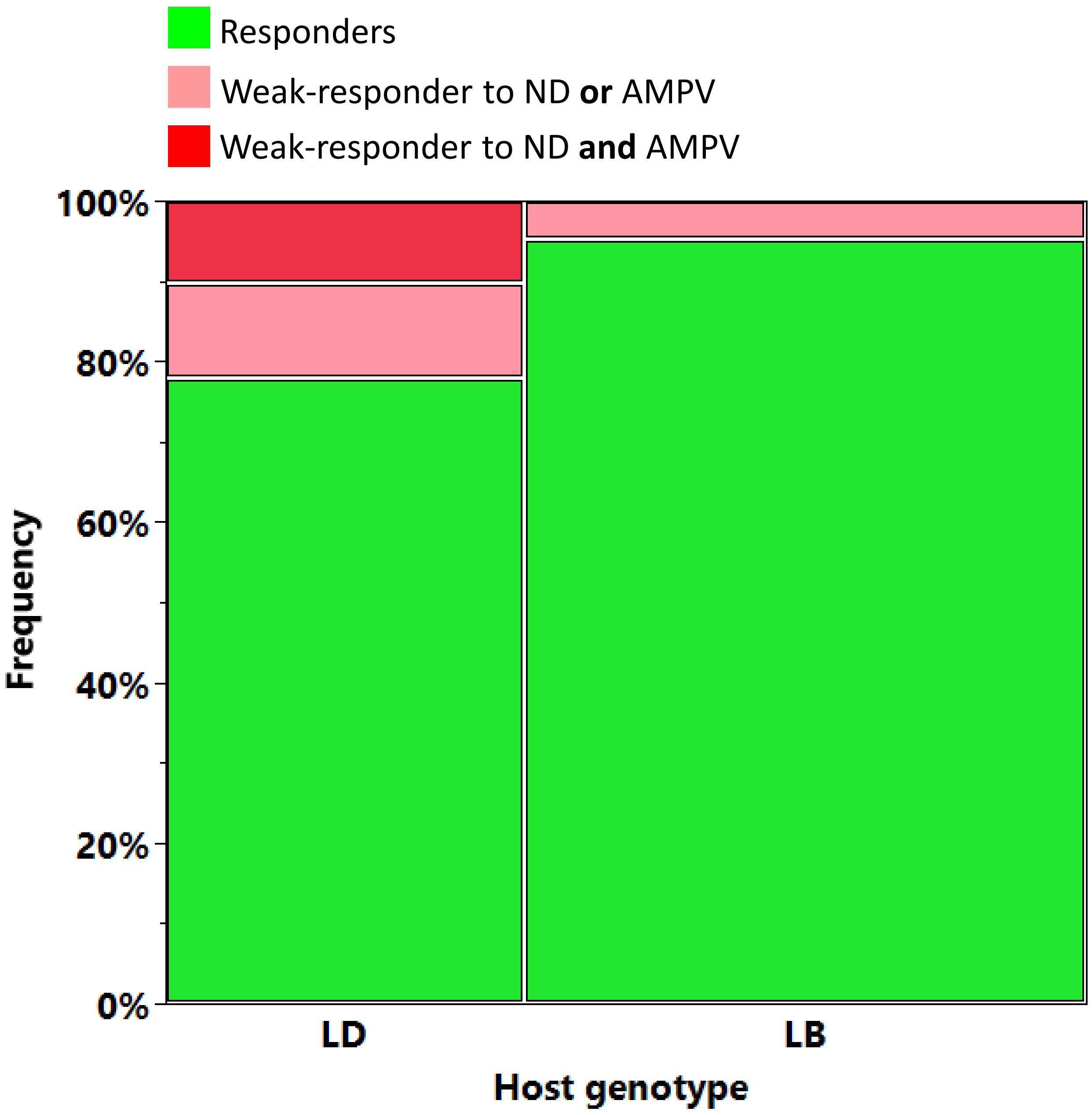

Supplementary Figures S1. Frequency of responder and weak-responder hens to vaccinations with ND and/or AMPV.
